# The Effect of Initial High vs. Low FiO_2_ on Breathing Effort in Preterm Infants at Birth: A Randomized Controlled Trial

**DOI:** 10.3389/fped.2019.00504

**Published:** 2019-12-12

**Authors:** Janneke Dekker, Tessa Martherus, Enrico Lopriore, Martin Giera, Erin V. McGillick, Jeroen Hutten, Ruud W. van Leuteren, Anton H. van Kaam, Stuart B. Hooper, Arjan B. te Pas

**Affiliations:** ^1^Department of Neonatology, Leiden University Medical Center, Leiden, Netherlands; ^2^Center Proteomics Metabolomics, Leiden University Medical Center, Leiden, Netherlands; ^3^The Ritchie Centre, Hudson Institute for Medical Research, Melbourne, VIC, Australia; ^4^Department of Obstetrics and Gynaecology, Monash University, Melbourne, VIC, Australia; ^5^Department of Neonatology, Emma Children's Hospital, Amsterdam UMC, University of Amsterdam, Amsterdam, Netherlands

**Keywords:** preterm infant, respiratory effort, breathing, oxygen, resuscitation

## Abstract

**Background:** Infants are currently stabilized at birth with initial low FiO_2_ which increases the risk of hypoxia and suppression of breathing in the first minutes after birth. We hypothesized that initiating stabilization at birth with a high O_2_ concentration, followed by titration, would improve breathing effort when compared to a low O_2_ concentration, followed by titration.

**Methods:** In a bi-center randomized controlled trial, infants <30 weeks gestation were stabilized at birth with an initial O_2_ concentration of 30 or 100%, followed by oxygen titration. Primary outcome was minute volume of spontaneous breathing. We also assessed tidal volumes, mean inspiratory flow rate (MIFR) and respiratory rate with a respiratory function monitor in the first 5 min after birth, and evaluated the duration of mask ventilation in the first 10 min after birth. Pulse oximetry was used to measure heart rate and SpO_2_ values in the first 10 min. Hypoxemia was defined as SpO_2_ < 25th percentile and hyperoxemia as SpO_2_ >95%. 8-iso-prostaglandin F2α (8iPGF2α) was measured to assess oxidative stress in cord blood and 1 and 24 h after birth.

**Results:** Fifty-two infants were randomized and recordings were obtained in 44 infants (100% O_2_-group: *n* = 20, 30% O_2_-group: *n* = 24). Minute volumes were significantly higher in the 100% O_2_-group (146.34 ± 112.68 mL/kg/min) compared to the 30% O_2_-group (74.43 ± 52.19 mL/kg/min), *p* = 0.014. Tidal volumes and MIFR were significantly higher in the 100% O_2_-group, while the duration of mask ventilation given was significantly shorter. Oxygenation in the first 5 min after birth was significantly higher in infants in the 100% O_2_-group [85 (64–93)%] compared to the 30% O_2_-group [58 (46–67)%], *p* < 0.001. The duration of hypoxemia was significantly shorter in the 100% O_2_-group, while the duration of hyperoxemia was not different between groups. There was no difference in oxidative stress marker 8iPGF2α between the groups.

**Conclusion:** Initiating stabilization of preterm infants at birth with 100% O_2_ led to higher breathing effort, improved oxygenation, and a shorter duration of mask ventilation as compared to 30% O_2_, without increasing the risk for hyperoxia or oxidative stress.

**Clinical Trial Registration:** This study was registered in www.trialregister.nl, with registration number NTR6878.

## Introduction

In the fetus, spontaneous breathing movements enable small volumes of liquid to move in and out of the airways. During apnea, the glottis is predominantly closed, restraining the efflux of lung liquid which is essential for normal lung growth and development by maintaining the lung in a distended state (1–3). After birth, the requirement for breathing movements changes to ventilation of the gas exchange regions of the lung, which necessitates that the liquid filling the lungs is replaced with air. However, after birth the glottis continues to function as it does in the fetus. It closes during apnea, is predominantly closed and opens only briefly during spontaneous breaths when the breathing pattern is unstable, but is predominantly open if the newborn has a stable breathing pattern (4–6).

While most preterm infants need respiratory support at birth because of their low muscle strength and structural and biochemical immaturity of the lung, support is mainly provided non-invasively instead of invasively to reduce the risk of lung and brain injury. However, successful application of non-invasive respiratory support requires an open upper airway in order for air to enter the trachea and aerate the lung. A closed glottis during apnea hampers the effectiveness of non-invasively applied positive pressure ventilation. This has been demonstrated by both animal (4) and human studies, with the latter showing that a non-invasively applied sustained inflation in preterm infants at birth was ineffective unless the infant was spontaneously breathing (5). Interventions aimed at improving effectiveness of non-invasive ventilation should therefore focus on stimulation of spontaneous breathing in order to open the glottis and enable success of non-invasive respiratory support.

One of the factors influencing breathing activity is oxygenation. Hypoxia is a potent inhibitor of fetal breathing movements before birth and this inhibitory effect persists for days-weeks after birth (7). As hypoxia is a likely common mechanism for apnea at birth, hypoxia should be avoided in order to enhance spontaneous breathing and opening of the glottis so that non-invasive respiratory support can be more effective. This, in turn, might reduce the need for intubation and mechanical ventilation, thereby reducing the risk of lung and brain injury.

At birth, oxygenation is largely determined by the surface area available for gas exchange within the lung, the gas diffusion distance and the partial pressure gradient for oxygen between alveoli and adjacent capillaries. It has now become clear that, during stabilization of very and extremely preterm infants in the delivery room, to compensate for inadequate lung aeration a higher fraction of inspired oxygen (FiO_2_) is required because the surface area for gas exchange is suboptimal. However, the 2015 versions of the international resuscitation guidelines recommend that resuscitation of very and extremely preterm infants should commence with a low FiO_2_ in order to reduce the risk of hyperoxia (8–10). This is because hyperoxia is associated with increased production of oxygen free radicals leading to tissue damage in infants with an immature antioxidant capacity (11). This initial low FiO_2_ should then be titrated based on the 25th percentile of the oxygen saturation target ranges defined by Dawson et al. (12). The downside of this approach is an increased risk of hypoxia with a potential negative effect on breathing effort. It has been shown that persisting hypoxemia (SpO_2_ < 80%) at 5 min after birth is associated with a higher risk of mortality and the development of intraventricular hemorrhage (13). This might be caused by hypoxia-induced respiratory depression, leading to a higher risk of intubation and mechanical ventilation. Although multiple trials have assessed the effect of initiating stabilization at birth with high vs. low initial FiO_2_ levels on clinical outcomes, to date, breathing effort has not been evaluated with regard to the initial FiO_2_ used at birth.

We aimed to evaluate the effect of reducing hypoxemia on breathing effort of preterm infants at birth by using an initial high vs. low FiO_2_ (1.0 vs. 0.3). We hypothesized that initiating resuscitation of preterm infants at birth with a high FiO_2_ will increase breathing effort.

## Methods

A single-blinded randomized controlled trial was conducted at the Leiden University Medical Centre (LUMC) and the Amsterdam University Medical Centre (Amsterdam UMC), both located in the Netherlands. Preterm infants with a gestational age between 24^0/7^ and 29^6/7^ weeks were included. Infants with congenital abnormalities or conditions that might have an adverse effect on breathing effort or ventilation were excluded. Infants were randomized for resuscitation with an initial FiO_2_ of 0.3 (30% O_2_-group) or 1.0 (100% O_2_-group) using Castor EDC (Amsterdam, the Netherlands), an electronic data capture system. Allocation was stratified by gestational age (24^0/7^-26^6/7^ weeks and 27^0/7^-29^6/7^ weeks), using variable block sizes (4–6).

After the initial FiO_2_ setting, FiO_2_ was titrated following SpO_2_ target ranges recommended by our local guidelines on stabilization after birth, which are based on the target ranges described by Dawson et al. (12). When SpO_2_ was below the 25th percentile of these reference ranges, FiO_2_ was titrated up to 0.5 and subsequently to 1.0 when the initial FiO_2_ setting was 0.3. In case of an initial FiO_2_ setting of 1.0, FiO_2_ was maintained at 1.0. When SpO_2_ exceeded 90%, FiO_2_ was titrated down to 0.5 and subsequently to 0.3 and 0.21 when the initial FiO_2_ setting was 1.0. If SpO_2_ exceeded 90% after initiation with FiO_2_ 0.3, FiO_2_ was titrated down to 0.21 directly. SpO_2_ values were consistently monitored, and in case SpO_2_ values were outside the range, FiO_2_ was titrated every 30 s. Other procedures used in this study are described thoroughly in the published study protocol (14). At birth, cord clamping was performed after 30 s if the infants were apneic and after 60 s if the infants were breathing. In both groups, respiratory support was provided using a Neopuff^TM^ infant T-piece resuscitator (Fisher & Paykel Healthcare, Auckland, New Zealand) or a Giraffe Star System (Anandic Medical Systems, Feuerthalen, Switzerland). Respiratory function monitoring (RFM) data were recorded during stabilization in the first 5 min after birth to assess respiratory effort, and RFM data were collected of the first 10 min after birth to assess duration of positive pressure ventilation and oxygenation. Blinding of data analysis was performed to increase validity of outcomes, without interfering with clinical practice by blinding the oxygen blender or changing the caregiver responsible for oxygen titration.

The RFM was used to measure inflation pressures, flow and tidal volumes. This was used to calculate minute volume, mean inspiratory flow rate (MIFR), and the total ventilation time. Gas flow in and out of the facemask or endotracheal tube was measured using a small variable resistance anemometer, which also measured airway pressure (Varflex Flow Transducer, Vyaire Medical, Yorba Linda, CA,USA). The flow signal was integrated to provide inspired and expired tidal volumes in real time, with the difference in volume providing the degree of leak from either the facemask or endotracheal tube. SpO_2_ and heart rate were measured using either a Masimo SET pulse oximeter (Masimo Radical, Masimo Corporation, Irvine, California, USA) or Nellcor^TM^ pulse oximeter (Covidien, Dublin, Ireland); the probe was placed around the right wrist or hand of the infant. Data on oxygenation and heart rates were extracted using a Philips Intellivue MP30 monitor (Philips, Eindhoven, the Netherlands). The FiO_2_ was measured using a portable oxygen analyzer AX300-I (Teledyne Analytical Instruments, CA, USA). The signals were digitized at 200 Hz using the NewLifeBox-R physiological recording system (Advanced Life Diagnostics, Weener, Germany) and all signals were recorded by the NewLifeBox Neo-RSD computer system (Advanced Life Diagnostics, Weener, Germany) supported by Polybench physiological software (Applied Biosignals, Weener, Germany). Pulmochart software (Applied Biosignals, Weener, Germany) was used to analyze primary and secondary outcomes.

The primary study parameter was breathing effort, expressed as average minute volume normalized for body weight in the first 5 min after birth. Area under the curve (AUC) of minute volume was calculated using the minute data of the first 5 min after birth. Other tidal breathing parameters that were assessed, were: respiratory rate, tidal volumes and MIFR in the first 5 min after birth as well as the duration of ventilation given via facemask in the first 10 min after birth. The incidence of breaths with tidal volumes >4 and >8 mL/kg were compared between the groups. We assessed Apgar scores at 1 and 5 min after birth. In addition, oxygenation of the infants was assessed by measuring SpO_2_ values in the first 10 min and determining the amount of time that infants were considered to be hypoxemic [SpO_2_ < 25th percentile of the target ranges defined by Dawson et al. (12)] or hyperoxemic (SpO_2_ > 95%). Average FiO_2_ given at specific time points after birth was assessed as well as the total exposure to O_2_ by measuring the AUC of FiO_2_ vs. time curve, calculated using values collected every 0.5 s.

The level of oxidative stress was assessed by measuring the level of 8-iso-prostaglandin F2α (8iPGF2α) at birth by using cord blood, and 1 and 24 h after birth using blood samples of the infant. A detailed description of sampling and the rationale for this marker are presented in the study protocol (14). The samples were analyzed using the same method as described previously by Mas et al. (15). Validation of the analysis has been described in the Supplementary Material. The demographic data collected were: gestational age, birth weight, gender, antenatal use of corticosteroids, mode of delivery, complications during pregnancy (pregnancy-induced hypertension (PIH), preterm prelabor rupture of membranes (PPROM), intra-uterine growth restriction (IUGR), proven intra-uterine infection [in this study defined as positive blood culture in infant <72 h after birth)] and maternal medication use. Short-term clinical outcomes were noted: intraventricular hemorrhage ≥ grade III, intubation during resuscitation or within 24 h after birth and death before hospital discharge.

In a recent study, the average minute volume of preterm infants over the first 100 breaths was 150 ± 70 mL/kg/min (16). The study of van Vonderen et al. (17) shows an 80% relative increase in respiratory drive when FiO_2_ was switched from 0.21 to 1.0, in the first minute after FiO_2_ was increased (from 134 to 240 mL/kg/min). However, since we investigate a FiO_2_ of 0.3 instead of 0.21 in the lower oxygen arm, we expected half of the effect (40% relative increase) in respiratory drive than reported by van Vonderen et al. (17). For this, a sample size of 44 infants would be needed (α of 0.05 and power (1–β) of 0.8, 2-sided). Anticipating a 10% fail of recording of physiological measurements, and an additional risk of withdrawal or decline of consent after the infant was included, 52 infants were recruited.

Statistical analysis was performed with SPSS software version 23.0 (IBM, Chicago, Illinois; 2012). Although allocation was stratified by gestational age, outcomes are analyzed and presented for both treatment groups as a whole, because of the relatively small sample size. Differences in parameters of breathing effort in the first 5 min after birth between the study groups were tested with a Student's *t*-test if the data were found to be parametric, or with Mann-Whitney U test when the data were non-parametric. The interaction of minute volume, tidal volumes and MIFR in the first 5 min after birth with time were analyzed with a linear mixed model, in which both study group (treatment) and time were taken in consideration. Other study parameters and basic characteristics were explored to test for differences between the study groups. Demographical data and data on clinical outcomes are shown for all infants who were randomized, including infants for whom the primary or secondary outcomes could not be assessed. In addition, these data are also shown separately for the group of infants in whom primary and secondary outcomes could be assessed. Data are presented as mean ± SD for parametric data, median (IQR) for non-parametric data and n (%) for categorical data. *P* < 0.05 were considered significant.

## Results

**Figure 1:**
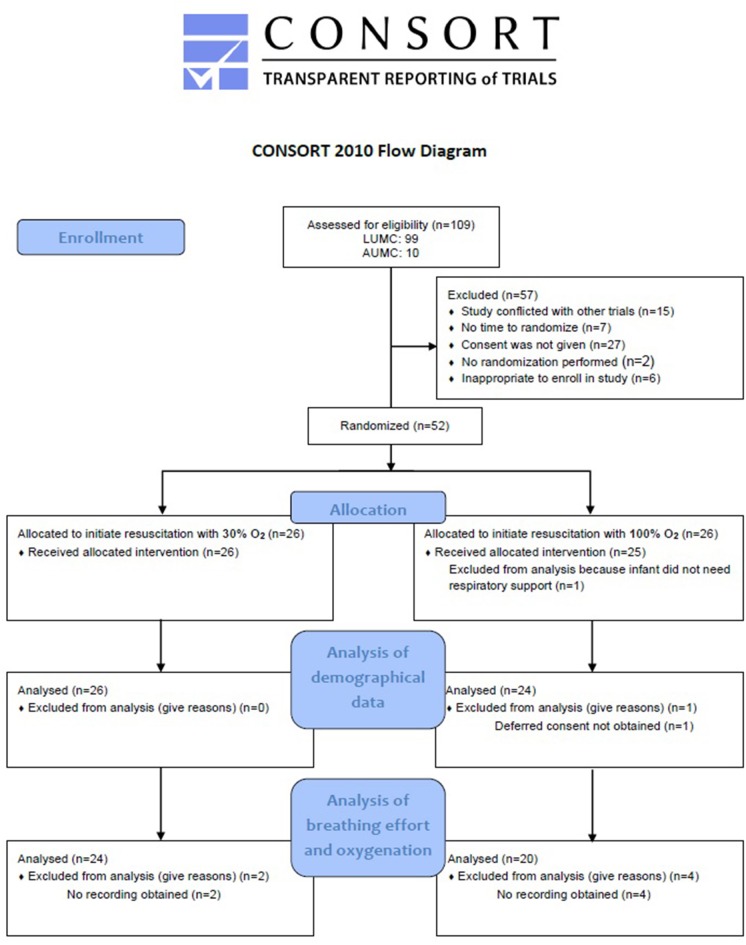
Flow diagram of allocation and analysis. LUMC, Leiden University Medical Centre; AUMC, Amsterdam University Medical Centre.

A total of 109 eligible infants were born in the LUMC and Amsterdam UMC during the study enrolment period between January 2018 and March 2019. Fifty-seven infants could not be included in this study because antenatal parental consent was refused, it was inappropriate to approach parents for considering enrolment in the study, the study was conflicting with other trials or because of technical issues with the RFM. Therefore, a total of 52 infants were randomized for initiation of resuscitation with either 100 or 30% O_2_. Six of these infants were excluded from analysis for outcomes on breathing effort and oxygenation due to failure to record physiological parameters with the RFM and pulse oximetry. One infant was excluded from the complete analysis because no respiratory support was needed and one infant because deferred consent was not obtained (Figure 1). In the 100% O_2_-group, 11 infants were recruited by prospective consent and nine infants by deferred consent, and in the 30% O_2_-group 11 infants were recruited prospectively and 13 infants with deferred consent.

There were no significant differences between the 100% and the 30% O_2_-group with regard to gestational age, gender, birth weight, percentage of infants that received a full course of antenatal steroids or mode of delivery in both the total group and the group where physiological parameters were recorded (Tables 1, 2). Neither were there any differences regarding maternal medication use or complications during pregnancy that could have had an effect on respiration of the infant at birth (Tables 1, 2).

**Table 1:** Demographical data patients randomized.

	**100% O_**2**_**	**30% O_**2**_**	***p*-value**
	***n* = 24**	***n* = 26**	
Gestational age (weeks)^a^	27^2/7^ ± 1^6/7^	27^1/7^ ± 1^4/7^	0.746
Birth weight (grams)^a^	1,000 ± 291	934 ± 258	0.405
Gender (% male)^b^	13/24 (54%)	9/26 (35%)	0.565
Mode of delivery (% cesarean section)^b^	14/24 (58%)	11/26 (42%)	0.396
Antenatal corticosteroids (% full course)^b^	11/24 (46%)	14/26 (54%)	0.606
Maternal medication use influencing respiration of the infant^b^	0/24 (0%)	0/26 (0%)	1.000
Complications during pregnancy^b^			0.082
PPROM	2/24 (8%)	2/26 (8%)	
PIH	4/24 (17%)	1/26 (4%)	
Intra-uterine infection	0/24 (0%)	2/26 (8%)	
IUGR	0/24 (0%)	5/26 (19%)	
Multiple	1/24 (4%)	0/26 (0%)	

a *Data are presented as mean ± SD for parametric data*.

b *n (%) for categorical data*.

*PPROM, preterm prelabor rupture of membranes; PIH, pregnancy-induced hypertension; IUGR, intra-uterine growth restriction*.

**Table 2:** Demographical data patients analyzed.

	**100% O_**2**_**	**30% O_**2**_**	***p*-value**
	***n* = 20**	***n* = 24**	
Gestational age (weeks)^b^	27^6/7^ (25^2/7^-28^6/7^)	27^2/7^ (25^4/7^-28^3/7^)	0.671
Birth weight (grams)^a^	992 ± 309	931 ± 256	0.481
Gender (% male)^c^	11/20 (55%)	17/24 (71%)	0.352
Mode of delivery (% cesarean section)^c^	12/20 (60%)	10/24 (42%)	0.364
Antenatal corticosteroids (% full course)^c^	9/20 (45%)	14/24 (58%)	0.628
Maternal medication use influencing respiration of the infant^c^	0/20 (0%)	0/24 (0%)	1.000
Complications during pregnancy^c^			0.227
PPROM	2/20 (10%)	2/24 (8%)	
PIH	3/20 (15%)	1/24 (4%)	
Intra-uterine infection	0/20 (0%)	2/24 (8%)	
IUGR	0/20 (0%)	5/24 (21%)	
Multiple	1/20 (5%)	0/24 (0%)	

a *Data are presented as mean ± SD for parametric data*.

b *Median (IQR) for non-parametric data*.

c *n (%) for categorical data*.

*PPROM, preterm prelabor rupture of membranes; PIH, pregnancy-induced hypertension; IUGR, intra-uterine growth restriction*.

**Figure 2:**
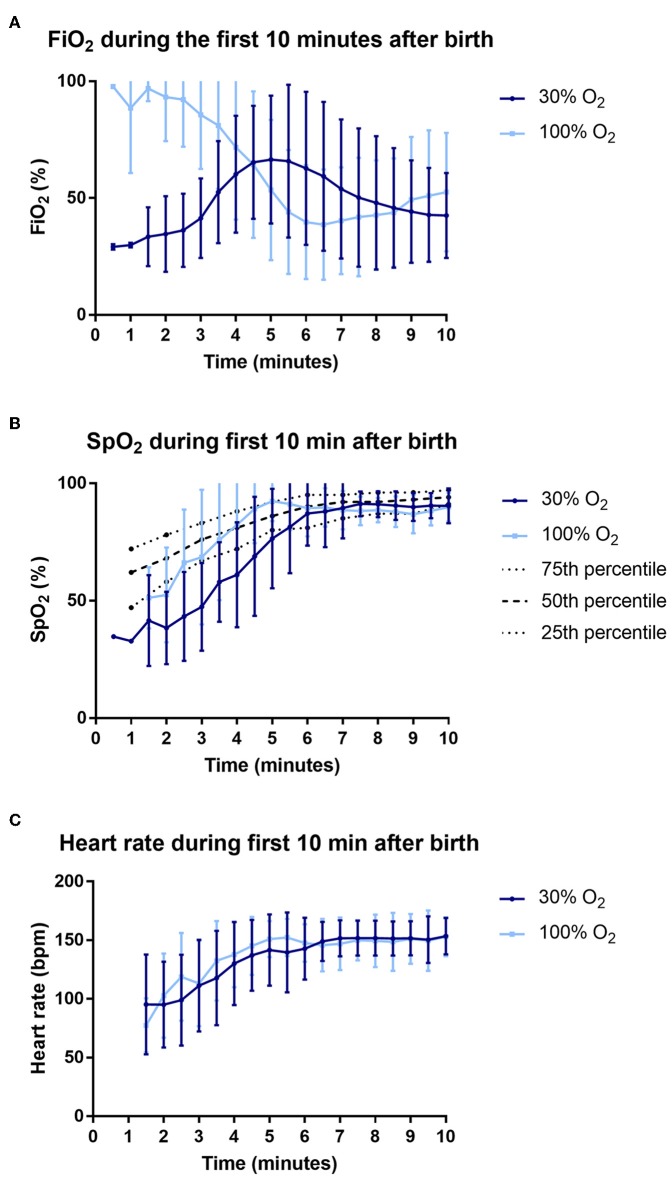
Data on fraction of inspired oxygen (FiO_2_) **(A)**, oxygen saturations (SpO_2_) **(B)** and heart rate **(C)** during the first 10 min after birth.

The average FiO_2_ received in the first 5 min after birth was significantly higher in the 100% O_2_-group compared to the 30% O_2_-group (FiO_2_ 0.91 (0.67–0.98) vs. FiO_2_ 0.48 (0.33–0.53), *p* < 0.001), as was the average FiO_2_ in the first 10 min after birth (FiO_2_ 0.69 (0.41–0.88) vs. FiO_2_ 0.45 (0.33–0.64), *p* = 0.020). Over the first 5 min after birth, total exposure to O_2_ (measured as AUC of FiO_2_ vs. time) was significantly higher in the 100% O_2_-group (AUC_0−5_ 559.2 (346.6–745.2)%·min vs. 346.3 (260.9–450.9)%·min, *p* = 0.002, but when assessed over the first 10 min after birth it was not different between groups (AUC_0−10_ 1011.3 (784.0–1191.7)%·min vs. 844.1 (741.0–1006.7)%·min, *p* = 0.178). This was because FiO_2_ was rapidly reduced in the 100% O_2_-group, whereas at the same time FiO_2_ levels in the 30% O_2_-group were increasing (Figure 2A).

### Breathing Effort

**Figure 3:**
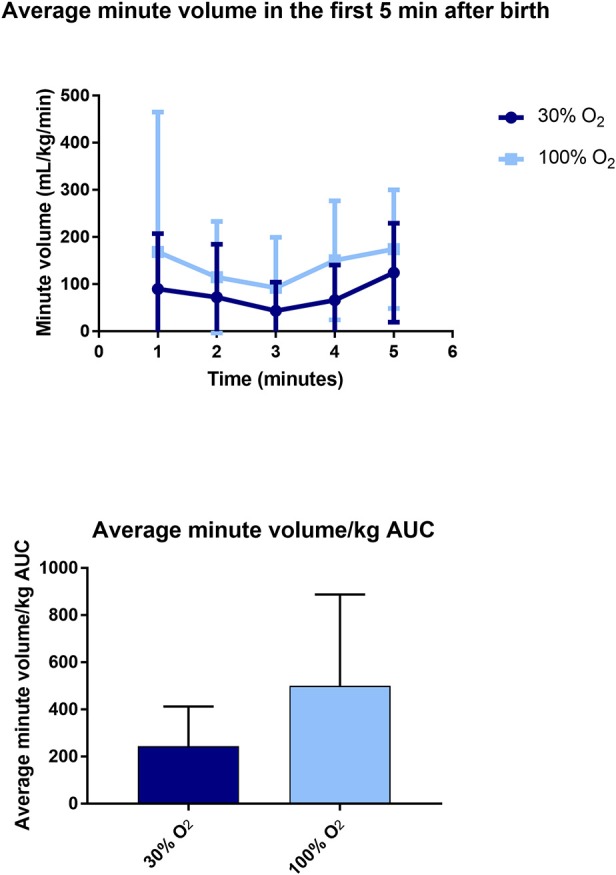
Data on average minute volume (upper) and area under the curve (AUC) of minute volume (lower) in the first 5 min after birth.

The minute volume averaged over the first 5 min after birth was significantly higher in the 100% O_2_-group compared to the 30% O_2_-group (146.34 ± 112.68 mL/kg/min vs. 74.43 ± 52.19 mL/kg/min, *p* = 0.014; Figure 3). Similarly, the cumulative minute volume measured over the first 5 min after birth (AUC of MV vs. time) was significantly higher in infants whose resuscitation commenced with 100% O_2_ (498.18 ± 390.10 min.mL/kg) compared with 30% (244.63 ± 168.42 min.mL/kg, *p* = 0.014; Figure 3).

**Figure 4:**
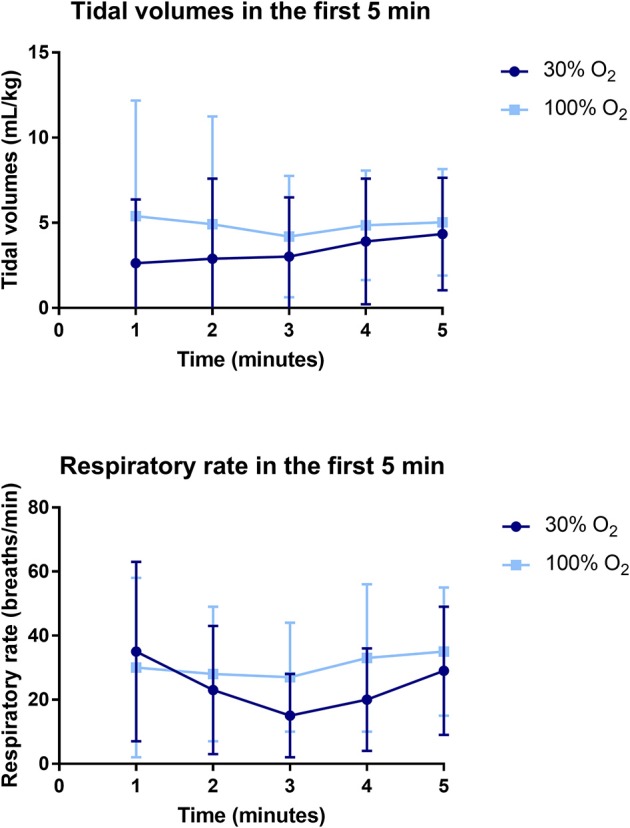
Data on tidal volumes (upper) and respiratory rate (lower) in the first 5 min after birth.

The average tidal volume and MIFR in the first 5 min after birth were significantly higher in the 100% O_2_-group (Table 3, Figure 4). When respiratory rates are measured at specific time points after birth, there was a trend toward a higher respiratory rate in the 100% O_2_-group, although this difference did not reach statistical significance (Table 3, Figure 4). Similarly, when the cumulative respiratory rate is measured over the first 5 min (AUC of respiratory rate vs. time) respiratory rates tended to be higher in the 100% O_2_-group (88.78 ± 58.54 vs. 69.83 ± 43.70 breaths, *p* = 0.226). Although there was a trend toward a higher incidence of breaths with a tidal volume >4 mL/kg (49 ± 25% vs. 35 ± 24%, *p* = 0.056), the incidence of recruitment breaths (>8 mL/kg) did not differ between study groups (Table 3).

**Table 3:** Parameters of respiratory effort.

	**100% O_**2**_**	**30% O_**2**_**	***p*-value**
	***n* = 20**	***n* = 24**	
Average tidal volumes in first 5 min (mL/kg)^a^	4.8 ± 3.8	3.8 ± 3.7	0.006
Average MIFR in first 5 min (mL/kg/s)^a^	12.7 (5.7–18.0)	7.8 (3.3–13.3)	0.014
Respiratory rate (breaths/min)^b^	33 (14-52)	26 (11-36)	0.099
Time of mask ventilation in the first 10 min (s)^b^	23.6 (0.0–122.2)	108.3 (46.4–205.1)	0.021
Incidence of breaths with tidal volume >4 mL/kg^a^	50 ± 24	35 ± 24	0.056
Incidence of recruitment breaths (>8 mL/kg)^b^	6 (1-24)	5 (0–18)	0.873

a *Data are presented as mean ± SD*.

b *Median (IQR) for non-parametric data*. *MIFR, mean inspiratory flow rate*.

The duration of mask ventilation was significantly shorter in the 100% O_2_-group [23.6 (0.0–122.2) s vs. 108.3 (46.4–205.1) s, *p* = 0.021]. While Apgar scores at 1 min after birth were not significantly different between the study groups [6 (3–8) vs. 5 (3–7), *p* = 0.149], infants in the 100% O_2_-group showed significantly higher Apgar score at 5 min after birth [8 (8–9) vs. 8 (7–8), *p* = 0.028], as a result of higher scores on breathing and tone.

### Oxygenation

The average oxygen saturation in the first 5 min after birth was significantly higher in infants in the 100% O_2_-group [SpO_2_ 85 (64–93)% vs. 58 (46–67)%, *p* < 0.001]. In addition, there was a trend toward a higher heart rate in the 100% O_2_-group (Table 4, Figure 2C). Infants in the 100% O_2_-group had SpO_2_ values between 90 and 95% for a significantly higher percentage of time in the first 5 min after birth, while at 5–10 min after birth this was not significantly different between the groups (Table 4, Figure 2B). A SpO_2_ of 80% was reached significantly earlier in the 100% O_2_-group (time 178 ± 70 s after birth vs. 261 ± 80 s after birth, *p* = 0.001). In addition, the duration of hypoxemia [SpO_2_ < 25th percentile of reference ranges described by Dawson et al. (12)] was significantly shorter in the 100% O_2_-group (Table 4). However, the duration of hyperoxemia (SpO_2_ > 95%) did not significantly differ between the study groups.

**Table 4:** Parameters of oxygenation.

	**100% O_**2**_**	**30% O_**2**_**	***p*-value**
	***n* = 20**	***n* = 24**	
Average SpO_2_ in first 5 min (%)^b^	85 (64–93)	58 (46–67)	<0.001
Average heart rate in first 5 min (bpm)^a^	134 ± 24	120 ± 28	0.086
Duration of hypoxemia (<25th percentile of Dawson target ranges) in first 10 min (s)^b^	73 (0–189)	158 (116–184)	0.018
Duration of hyperoxia in first 10 min (s)^b^	99 (24-215)	79 (15-152)	0.394
Percentage of time of SpO_2_ 90–95% in first 5 min (%)^b^	15 (2-38)	1 (0–9)	0.002
Percentage of time of SpO_2_ 90–95% in 5–10 min (%)^b^	30 (21-42)	35 (19-58)	0.556
Incidence of intubation in delivery room^c^	0/20 (0%)	2/24 (8%)	0.493

a *Data are presented as mean ± SD for parametric data*.

b *Median (IQR) for non-parametric data*.

c *n (%) for categorical data*.

*SpO_2_, oxygen saturation*.

### Oxidative Stress

**Figure 5:**
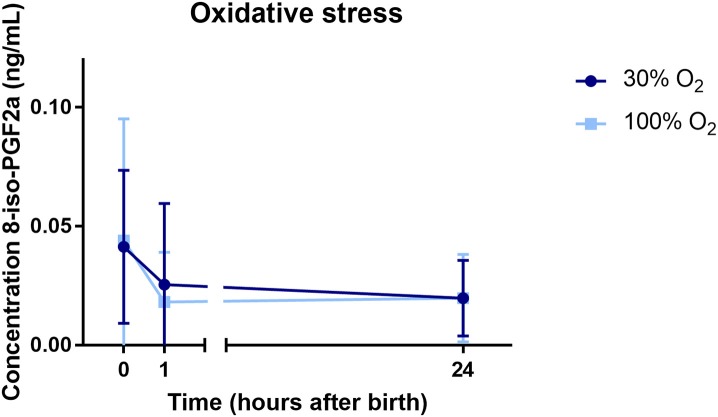
The concentration of 8-iso-prostaglandin F2α (8-iso-PGF2α) in the first 24 h after birth.

Cord blood from 32/44 (73%) infants could be obtained to analyze the level of 8iPGF2α at baseline. At 1 h after birth, blood samples of a total of 27/44 (61%) infants were obtained and analyzed, and at 24 h after birth, again blood samples of 25/44 (57%) infants were obtained and analyzed. The level of 8iPGF2α did not differ between the study groups over time, neither did the level of 8iPGF2α differ at the specific time points analyzed (Figure 5).

### Short-Term Clinical Outcomes

Only two infants of the total study population needed intubation in the delivery room, both infants were randomized to the 30% O_2_-group. There were no differences between the study groups with regard to intubation, incidence of IVH ≥ grade III or death before hospital discharge (Table 5).

**Table 5:** Short-term clinical outcomes.

	**100% O_**2**_**	**30% O_**2**_**	***p*-value**
	***n* = 24**	***n* = 26**	
Incidence of intubation in the delivery room	0/24 (0%)	2/26 (8%)	0.491
Incidence of intubation <24 h after birth	8/24 (33%)	7/26 (27%)	0.760
Incidence of IVH ≥ grade III	0/24 (0%)	4/26 (15%)	0.111
Incidence of death before hospital discharge	2/24 (8%)	5/26 (19%)	0.420

*Data are presented as n (%). IVH, intraventricular hemorrhage*.

## Discussion

In this bi-center, single blinded, randomized controlled trial, the effect of initiating resuscitation with an FiO_2_ of either 1.0 or 0.3 on breathing effort of preterm infants was evaluated. We demonstrated significantly higher breathing effort in preterm infants who were stabilized at birth with an initial FiO_2_ of 1.0 as compared to infants initiating with an FiO_2_ of 0.3. Similarly, oxygenation was significantly higher over the first 5 min after birth, with a shorter duration of hypoxemia without an increase in hyperoxemia or oxidative stress. While total O_2_ exposure (AUC of FiO_2_ vs. time) was increased over the first 5 min, it was less during the second 5 min in the 100% O_2_-group, resulting in no difference between groups after 10 min. The improved oxygenation and breathing effort were also reflected by a shorter duration of mask ventilation in the 100% O_2_-group. These results indicate that initiating stabilization at birth with a high FiO_2_ followed by careful titration to avoid hyperoxia, is a better option for stimulating breathing and decreasing the need for positive pressure ventilation in very and extremely preterm infants.

The effectiveness of non-invasive ventilation is hampered by a closed glottis during apnea, preventing air from entering the lung. While non-invasive respiratory support is universally adopted as the primary mode of respiratory support for preterm infants at birth, closure of the glottis between breaths underscores the importance of stimulating spontaneous breathing to enhance the success of this approach. Evaluating the effect of interventions used in the delivery room on breathing effort of preterm infants is therefore important to guide clinicians in prioritizing interventions to increase the effectiveness of non-invasive respiratory support. Although multiple randomized trials have been performed comparing the effect of a high vs. low initial FiO_2_ during stabilization of preterm infants at birth, none of these trials have evaluated breathing effort (18–23). The effect of oxygenation on breathing effort has so far only been demonstrated in an observational study where an increase in breathing effort was observed after switching FiO_2_ from 0.21 to 1.0 (17). However, we recently demonstrated the effect of the use of a FiO_2_ of 1.0 at birth on breathing effort in a spontaneously breathing preterm rabbit model (24). When compared to stabilization with FiO_2_ 0.21, an FiO_2_ of 1.0 led to a more stable breathing pattern with a lower variability in inter-breath interval, higher respiratory rate and less apnea. We have now observed similar results in preterm infants, despite the fact that oxygen was titrated down instead of using a continuous FiO_2_ of 1.0, as used in preterm rabbits. This indicates that the initiating stabilization at birth with a high FiO_2_ reduces the hypoxia-induced respiratory depression, after which a reduction in FiO_2_ does not impact breathing effort.

The effect of other interventions used to increase breathing effort in the delivery room has been described previously (25, 26). Application of a regimented protocol of repetitive tactile stimulation to preterm infants at birth resulted in clinically relevant (although not statistically significant) increase of breathing effort, as compared to stimulation at the discretion of the caregiver; the result was not significant due to an increase in stimulation in the control group compared with historical levels of stimulation (25). In addition, infants who have been administered caffeine in the delivery room showed a significantly higher breathing effort (26). However, the magnitude of the difference in minute volume we found between the 100 and 30% O_2_-groups in the current study is much greater than the effect of any other treatment previously investigated in preterm infants at birth. This indicates that oxygen might be a major contributor to improving breathing effort at birth.

Infants in the 100% O_2_-group had significantly higher oxygenation levels (higher SpO_2_ values) and shorter periods of hypoxemia than infants in the 30% O_2_-group. Apart from the initial FiO_2_, our intent was to manage all infants with the same respiratory support strategy, but the 100% O_2_-group required less intermittent positive pressure ventilation than the 30% O_2_-group. As such, we consider that the higher initial partial pressure gradient for oxygen is likely to be the major contributor to the improved oxygenation in the 100% O_2_-group. While the total O_2_ exposure (AUC of FiO_2_ vs. time) was increased over the first 5 min after birth, it was not different between groups when measured over the first 10 min after birth. This is because the FiO_2_ was reduced between 2 and 6 min after birth in the 100% O_2_ group, whereas over the same time period, the FiO_2_ increased in the 30% O_2_ group (Figure 2). The finding that saturations were increasing while the FiO_2_ levels were decreasing in the 100% O_2_ group (between 2 and 6 min after birth) indicates that the O_2_ exchange capacity of the lung markedly increased over this time. This finding supports our contention that higher partial pressure gradients for oxygen (i.e., higher FiO_2_ levels) should be used initially, when the lung is partially aerated and the surface area for gas exchange is low, and that the FiO_2_ can be rapidly titrated down as the lung aerates and the potential for oxygen exchange increases. Indeed, as lung aeration and the increase in gas exchange surface area occurs in an exponential manner, presumably the decrease in FiO_2_ can follow a similar function.

Previous trials comparing the use of high vs. low initial oxygen concentrations for supporting preterm infants at birth show conflicting results. Some trials have found that initiating resuscitation with a low FiO_2_ (<0.3) results in significantly lower SpO_2_ values compared to a high FiO_2_ (>0.9) (18, 20, 23), whereas other trials detected no significant differences in oxygenation (19, 21, 22). However, different FiO_2_ titration protocols (both increasing and decreasing) between studies are likely to be a major confounding factor. FiO_2_ titration steps have ranged between 0.1 and 0.79, with step durations ranging between 10 s to every 5 min. There is also much heterogeneity in timepoints at which outcomes were assessed, with measurement start times ranging from at birth to 2 min after birth, whereas measurement end times ranging from 5 to 30 min after birth. Probably just as important is the prevention of hyperoxia and the tissue damage caused by increased oxygen free radicals. In our study, we found no difference in the duration of hyperoxemia or in the level of oxidative stress (assessed by measuring 8iPGF2α levels) between infants initially receiving either 30% or 100% O_2_. However, a previous study found that oxidative stress markers (advanced oxidative protein products AOPP and 8iPGF2α) were increased at 2 and/or 12 h after birth in infants initially receiving a high (FiO_2_ 1.0) vs. low (FiO_2_ 0.21) FiO_2_ (To2RPIDO trial) (27). Again, a difference in titration steps and timepoints of assessing the outcomes could explain the difference between these results and the results of our trial. The current study was not powered to detect differences in the level of oxidative stress, and in addition, blood samples could not be obtained and analyzed from every included infant. However, the results are from a large random sample and are likely representative for the total cohort.

Improving oxygenation in the first 5 min after birth has gained increased attention, as it has become clear that persistent hypoxemia at >5 min after birth is associated with an increased risk of intraventricular hemorrhage and mortality (13). As hypoxia inhibits breathing both in the fetus as well as after birth, persistent hypoxia likely reduces the efficacy of non-invasive ventilation resulting in an increased need for intubation and mechanical ventilation (7). Our trial was not designed to detect significant differences in clinical outcomes. However, it should be noted that IVH and death before hospital discharge occurred non-significantly more often in the 30% O_2_-group [IVH 0/24 (0%) vs. 4/26 (15%), *p* = 0.111; death before hospital discharge 2/24 (8%) vs. 5/26 (19%), *p* = 0.420].

The recent systematic review including trials on high vs. low FiO_2_ during resuscitation of preterm infants at birth showed no difference in clinical outcomes, including mortality, neurodevelopmental impairment and other key preterm morbidities (14). However, the level of evidence of included studies was assessed to be very low. In addition, the randomized trials included in this meta-analysis were highly variable with regard to the gestational age of included infants (<28– <35), FiO_2_ levels used for high (ranging from 0.6 to 1.0) and low (0.21–0.5) FiO_2_ study groups and SpO_2_ targets. In our study, we aimed to provide knowledge on one of the factors that could contribute to improve breathing effort at birth. It was shown that oxygen is a major contributing factor, in addition to other factors that have previously been shown to increase breathing effort, such as tactile stimulation and caffeine administration in the delivery room (25, 26). Incorporating these factors into the resuscitation guidelines on delivery room management may increase our effectiveness of supporting preterm infants non-invasively, leading to less intubation and mechanical ventilation and better clinical outcomes. We have demonstrated that oxygenation is a major determinant to enhance respiratory effort, but careful titration, which is mindful of the exponentially increasing potential of the lung to exchange oxygen, is required to prevent hyperoxia. It is time to rethink our current strategy of supporting preterm infants at birth, during which we allow them to be hypoxemic in the first minutes after birth. As long as we do not have found the best way to aerate the lung, we would need a higher oxygen concentration at birth to compensate for partial lung aeration at birth, in order to improve oxygenation and stimulate spontaneous breathing. Again, this has to be followed by careful titration of oxygen to reduce the risk for hyperoxia.

## Conclusion

In this randomized controlled trial, we have demonstrated significantly higher breathing effort when resuscitation was initiated with an O_2_ concentration of 100% compared to 30% in very and extremely preterm infants. In addition, infants in the 100% O_2_-group were significantly better oxygenated with a shorter duration of hypoxemia without increasing the duration of SpO_2_ >95%, the total oxygen exposure (as measured by area under the curve of FiO_2_ received in the first 10 min after birth) or the level of 8-iso-prostaglandin F2α, measured at 1 and 24 h after birth. The results suggest that oxygenation might be an important determinant in stimulating breathing and decreasing the need for positive pressure ventilation in preterm infants at birth.

## Data Availability Statement

The datasets generated for this study are available on request to the corresponding author.

## Ethics Statement

The ethical committees of the LUMC and Amsterdam UMC approved the study protocol. Informed parental consent was obtained antenatally when possible. In case of an emergency situation (e.g., mother in full labor or when immediate delivery was necessary) or when obtaining antenatal consent was inappropriate (e.g., if the condition of the mother did not allow for proper consideration on participation), consent was asked retrospectively.

## Author Contributions

JD, EL, MG, SH, and AP made substantial contributions to conception and design of the study. JD, TM, EL, EM, JH, RL AK, and AP recruited and included infants in the study and obtained data. Data was analyzed and interpreted by JD, TM, MG, AP, and SH. The first version of the manuscript was drafted by JD, AP, and SH. All authors are acknowledged for their critical revision of the manuscript and approval of the final version.

### Conflict of Interest

The authors declare that the research was conducted in the absence of any commercial or financial relationships that could be construed as a potential conflict of interest.
